# The C repressor of the P2 bacteriophage

**DOI:** 10.1007/s10858-015-9999-3

**Published:** 2015-12-22

**Authors:** Tariq Massad, Evangelos Papadopolos, Pål Stenmark, Peter Damberg

**Affiliations:** Department of Biochemistry and Biophysics, Stockholm University, 10691 Stockholm, Sweden; Department of Biological Chemistry and Molecular Pharmacology, Harvard Medical School, 240 Longwood Av, Boston, MA 02115 USA; Department of Clinical Neurosciences, Karolinska Institute, 171 77 Stockholm, Sweden; Karolinska Research and Imaging Center, Karolinska University Hospital, 171 76 Stockholm, Sweden

## Biological context

The C-repressor of bacteriophage P2 (P2 C) is a DNA-binding protein that controls the lifecycle of the P2 bacteriophage by directing it toward the lysogenic mode. P2 C is a 99 amino acids protein, which forms stable homodimers but not higher oligomers in the absence of DNA (Ahlgren-Berg et al. 2007). As opposed to the more common situation where dimeric proteins bind palindromic DNA-sequences, P2 C binds cooperatively to two direct repeats of DNA (named O1 and O2) flanking the −10 region in the Pe promoter in the genetic switch of the bacteriophage P2. The two 8 basepairs long direct repeats have a centre-to-centre distance of 22 base pairs (Ljungquist et al. 1984). According to a Electrophoretic Mobility Shift Assay (EMSA) analysis (Ahlgren-Berg et al. 2007), P2 C induces a high degree of bending of DNA upon binding. The puzzling question how a symmetric protein dimer can bind to an asymmetric DNA binding site where the epitope is repeated twice, as opposed to the more common inverted repeats. To the best of the authors’ knowledge, there are only three other examples of proteins that bind direct repeated DNA sequences in the protein databank. Those are the λ-CII (Jain et al. 2005), the ω-repressors (Weihofen et al. 2006) and the mammalian HOT1 (Kappei et al. 2013), which are all structural different to P2C (Massad et al. 2010). The DNA-binding epitope of P2 C is located in the N-terminus (residues 1–54), which contains a helix-turn-helix (HTH) motif (Eriksson et al. 2000; Massad et al. 2010). It has been reported that upon the superinfection of the satellite bacteriophage P4 of a P2 lysogenic cell, P4 is able to derepress the P2 lysogen (Liu and Haggård-Ljungquist 1999). This is mediated by binding the P4 E antirepressor to the P2 C after infection leading to the formation of multimeric complexes, thereby preventing the P2 C from binding to its operator (Liu and Haggård-Ljungquist 1999).

Several mutations have been done on P2 C combined with activity assays to study the C-termini, the dimerization interface and the HTH motif, and to study the deactivation of P2 C by the P4 E antirepressor (Eriksson et al. 2000; Massad et al. 2010). One of the most interesting mutations is the truncation mutation performed on the last 9 residues of the C-terminus, which proved that the P2 C is still active even after truncation, indicating that the C-terminus might not be directly involved in the interaction with DNA. Solving the 3D structure of P2 C improves our understanding of its function and it is the first step to determine its DNA-binding mode.

The backbone assignment of the P2 C has been published and deposited in the Biological Magnetic Resonance Bank (BMRB) under accession code 15577 (Massad et al. 2008). Here we report the solution structure of the P2 C together with the order parameters calculated from ^15^N relaxation data using the model-free approach. We have previously reported the crystal structure (PDB 2XCJ) of P2 C at 1.8 Å (Massad et al. 2010), where P2 C was shown to be in a homodimeric state. The crystal structure indicated five rigid helices in the N-terminus and a β-turn in the C-terminus. Since P2 C is a homodimeric protein in the absence of DNA, its dimer interface in solution has been determined with aid from the crystal structure assuming no conformational changes during the crystallization process.

## Methods and results

An *E. coli* strain BL21(DE3) containing plasmid pEE679 expressing P2 C was grown at 310 K in M9 minimal medium containing ^13^C labeled-glucose, ^15^N labeled-NH_4_Cl and ampicillin (100 mg/ml) for 6–8 h until an OD_600_ = 0.6 was reached. Protein expression was induced by addition of isopropyl β-d-thiogalactoside (IPTG) to a final concentration of 1 mM at 37 °C for 4 h. The cells were harvested by centrifugation for 20 min at 9,000*g* at 4 °C and resuspended in 10 mM sodium phosphate buffer, pH 7.0. Cells were lysed by freezing/thawing together with sonication and thereafter centrifuged at 31,000*g* for 15 min at 277 K. The supernatant was collected and filtered with a 0.45 μm filter before starting the purification process. The protein was purified using ÄKTA™ FPLC-system in three consecutive steps. First, the filtered sample was adjusted to pH 8.0 with 5 M NaOH and loaded on a weak anion exchange column (DEAE, GE Healthcare) that had been equilibrated with 10 mM sodium phosphate buffer, pH 7.0 (running buffer). P2 C elutes with the flow through, as the pH of the running buffer is lower than the pI of P2 C. The second step was affinity chromatography using a HiTrap Heparin HP column equilibrated with running buffer. P2 C was eluted by a nine-column volume gradient of 1 M NaCl. The eluted fractions contain P2 C were loaded on a Superdex 200 gel filtration column (GE Healthcare) for further purification using 10 mM Na-Phosphate buffer, pH 7.0, 150 mM NaCl as running buffer. Finally, the sample was concentrated to 6 mg/ml using Amicon Ultra-15 centrifugal tubes (Millipore) with molecular weight cutoff 5 kDa. D_2_O was added to a final concentration of 10 % before the protein was transferred into a 5 mm NMR tube.

In order to obtain the NOE constraints, the 3D ^13^C-NOESY-HSQC and ^15^N-NOESY-HSQC (Zhang et al. 1994) datasets with 150 ms mixing time each where acquired at 310 K using Varian INOVA 900 MHz at the Swedish NMR Centre, Göteborg, Sweden. Standard ^13^C filtered NOESY experiments were performed to determine the dimer interface where ^15^N, ^13^C labeled P2 C was mixed with a nonlabeled variant. Those experiments failed possibly due to insensitivity of the experiments, the size of the protein or unfavorable exchange times, i.e. if the final concentration of mixed labeled:unlabeled dimers was too low. Spectra were processed using NMRPipe (Delaglio et al. 1995) and analyzed using Sparky (Goddard and Kneller). Model structures were generated using CYANA2.1 software (Güntert 2004). TALOS (Cornilescu et al. 1999) was utilized to generate empirical dihedral angles constraints for 75 residues based on the sequence and chemical shift assignment. A total of 1791 NOEs were manually assigned. The dimer interface of the crystal structure was examined for potential inter domain NOEs. The interdomain pairs M66-methly-M50H^γ,^ L63H^γ^-T62-methyl, A71methyl-S74H^β^, I5methyl-D47-H^β^ & T67H^γ^-T59H^γ2^ were identified as connectivities, which would display intense interdomain NOEs but not intradomain NOEs based on the distance. Out of the 1791 manually assigned NOEs those five were specified as interdomain, while the other were treated as ambiguous in the first CYANA iteration. A total of 100 structures were generated for each of the seven CYANA iterations and best structures were selected for the next iteration. Through network anchoring algorithm CYANA identified 3887 intradomain NOEs, as well as 128 interdomain NOEs, where the latter where all manually verified by examining the spectra to ensure reliability. Table 1 shows the structural statistics of P2 C.

**Table 1:** Structural Statistics for P2 C

Average target function (A^2^)	2.9 ± 0.18
*Upper distance limits*	
Total	4015
Short-range, |i − j| ≤ 1	1653
Medium-range, 1 < |i − j| < 5	1043
Long-range, |i − j| ≥ 5	1191
Interdomain	128
*Average RMSD to mean*	
Backbone	0.34 ± 0.12
Heavy atoms	0.78 ± 0.1
*PROCHECK statistics*	
Number of conformers	20
Restraints per residue	24.6
RMS deviation for bond lengths	0.001 Å
RMS deviation for bond angles	0
Average of bad steric contacts/100 residues	0
*Ramachandran quality (1*–*81)*	
Residues in most-favored regions	85 %
Residues in additionally allowed regions	15 %
Residues in generously allowed regions	0 %
Residues in disallowed regions	0.1 %

**Fig. 1:**
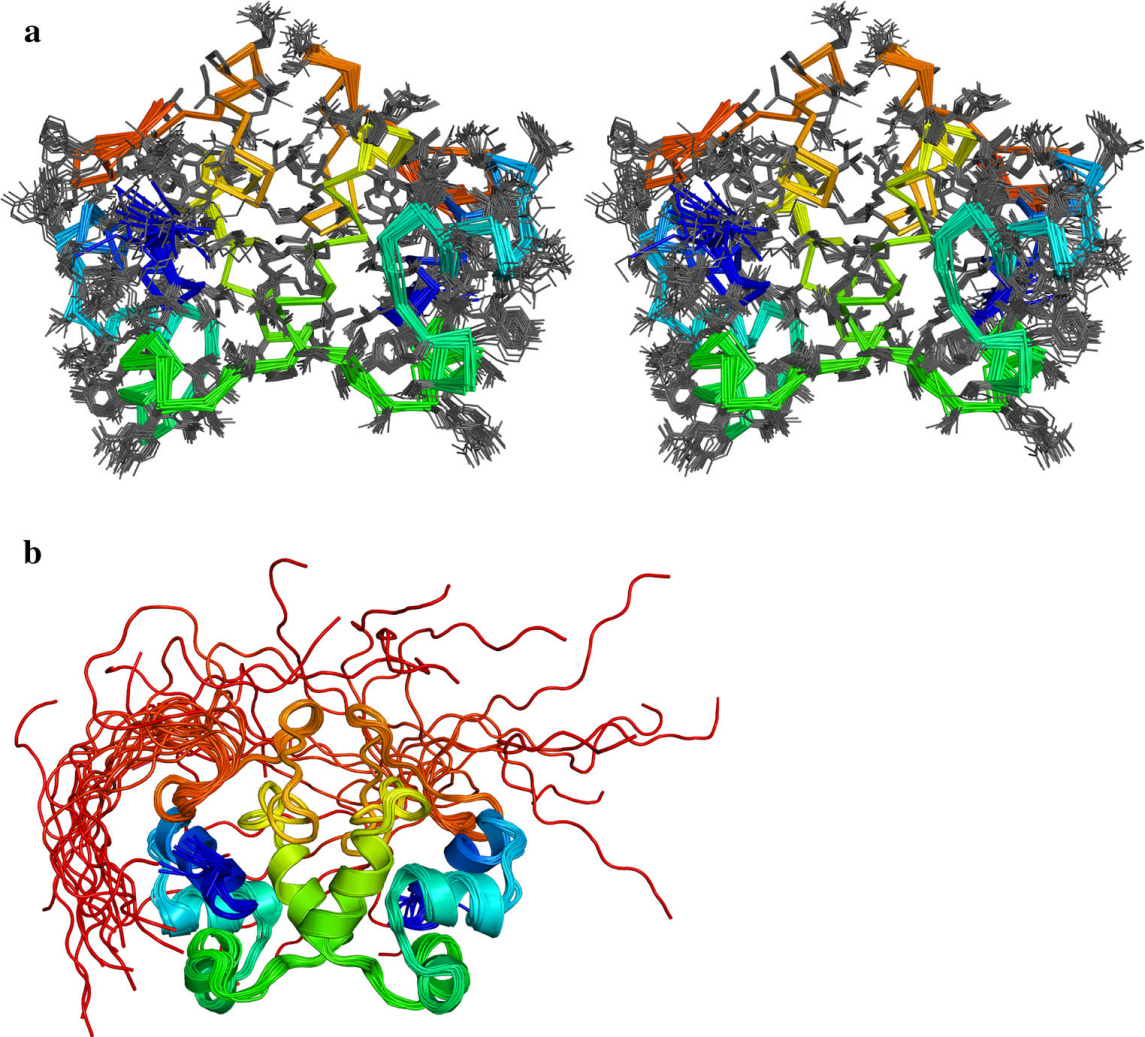
Rainbow colored overlays of the backbone of the 20 conformations of lowest target function with blue N-termini and red C-termini. **a** Stereo views of Ca-trace and heavy atoms from the side-chains of residues 1–85 **b** ribbon representation illustrating the flexible C-termini

The solution structure of P2 C reveals five short α-helices in each monomeric unit; I5-E16 (helix 1), R20-T26 (helix 2), Y31-S39 (helix 3), T46-Q54 (helix 4), Q57-M66 (helix 5) and a β-sheet-like structure made up by residues Q69-Q76. The C-terminus (starting from residue H85) is shown to be fully flexible and unstructured from the chemical shift index and ^15^N-relxation data. Figure 1 shows stereo views of the ensemble of structures with lowest target function.

## Backbone dynamics of P2 C

**Fig. 2:**
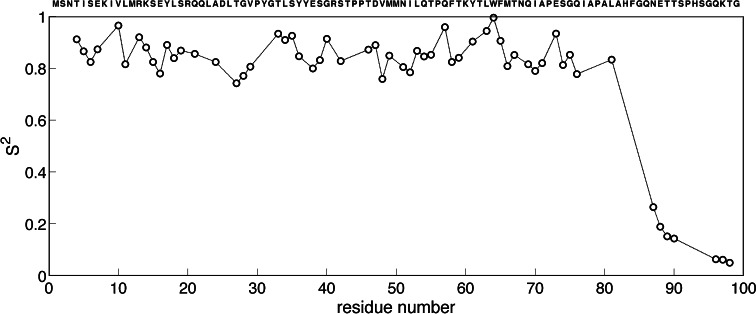
The generalized order parameter S^2^ of P2 C. The average S^2^ value for the C-terminus (residues 5–85) is 0.85 corresponding to rigid segments. The N-terminus displays values corresponding to highly flexible residues

The internal flexibility of P2 C was investigated by measuring ^15^N T1, ^15^N T2 and ^15^N-H NOE relaxation data at a 700 MHz magnetic field (CREM, Florence, Italy). The model-free formalism was used to analyze the dynamics assuming isotropical tumbling with Δσ = 169, r_H–N_ = 1.02 Å (Damberg et al. 2005) and an overall correlation time of 13.5 ns, as calculated for a 22 kDa globular protein was used in the analysis. The model-free fits were generated using FastmodelFree software (Cole and Loria 2003). The generalized order parameter S^2^ is successfully calculated for 69 residues that show intense well-resolved ^15^N-HSQC peaks. The ^15^N relaxation data shows that the core of the protein (residues 5–85) is rigid with a mean S^2^ = 0.85. The C-terminus (residues 87–99) of the protein has much lower S^2^ values ranging from 0.26 to 0.048 which is an indication of considerable flexibility, while the short turns connecting the α-helices and the β-sheet like structure are rigid on the ns-ps timescale (Fig. 2).

## P2 C-DNA complex

**Fig. 3:**
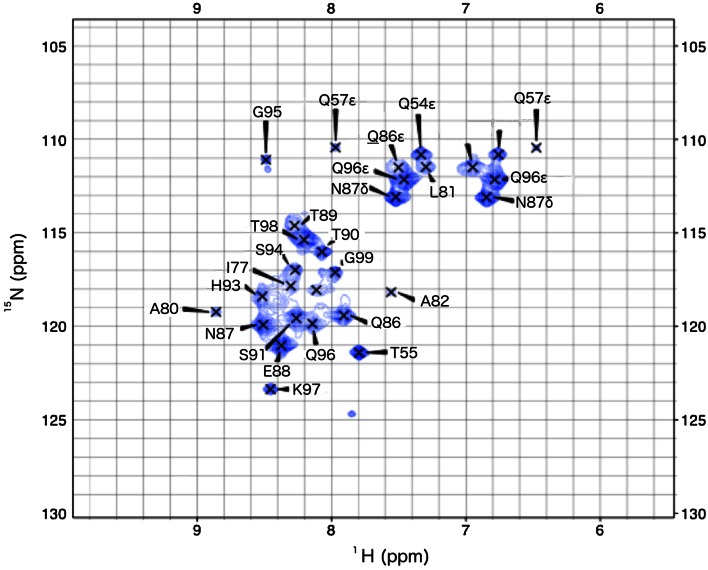
^15^N-HSQC of P2 C DNA Complex. Only peaks corresponding to the flexible c-terminus are observed in the spectrum

Wildtype ^15^N-labeled P2 C in NMR buffer (10 mM Sodium Phosphate, pH = 6.0) was mixed with a 42 bp piece of double stranded DNA with the sequence CATGGT**GTTTAGAT**CTCAATAGTATTTA**GTTTAGAT**GTAGAT and the complementary strand (Sigma) contains both O1 and O2 half sites (underlined and bold) at 278 K for 60 min. The molar ratio between P2 C and the DNA was 4 monomers: 1 stretch of DNA. The mixture was injected into a 3 mm NMR tube and D_2_O was added to a final concentration of 10 % and the concentration of double stranded DNA and monomeric protein was 50 and 200 μM, respectively. ^15^N-HSQC was recorded on an 800 MHz Bruker AVANCE spectrometer at 298 K, equipped with a *cryo* probe. The spectrum was processed with Topspin 2.1 (Bruker). The ^15^N-HSQC spectrum of the complex (Fig. 3) contained 29 peaks, which are tentatively assigned based on similarity of chemical shifts. The most C-terminal residues (85–99) display signals.

## Discussion and conclusions

We have determined the solution structure of the dimeric P2 C repressor protein. The solution structure of P2 C displays close agreement with the crystal structure with a backbone RMSD of 1.16 Å between the crystal structure and the NMR-structure with the lowest CYANA target function for residues 5–81. This is crucial for the structure calculations since the five manually assigned inter domain NOEs were inferred based on shorter inter atomic distances for inter domain pairs. If the crystal and solution structures were not similar those five assignments would potentially be incorrect. Incorrect inter domain constraints would guide the structure calculation towards an incorrect local minimum of the target function. In addition to the a priori argument for similarity between the crystal and solution structures, such as secondary chemical shifts, ^15^N-relaxation data and mutation studies, a large body of evidence accumulates during the structure calculation. In particular more than 4000 NOEs including more than 100 inter domain and the convergence to a tight ensemble of structures strikingly similar to the crystal structure support the initial assumption.

The final 20 lowest target function structures were evaluated using PROCHECK_NMR (Morris et al. 1992). All dihedral angles for structured residues are in allowed conformations of Ramachandran map (Lovell et al. 2003

The C-terminus is shown to be flexible in solution from NMR data with very low order parameters, random coil chemical shift index and the absence of NOESY peaks. In the crystal structure no electron density is observed for the C-terminal residues after G85, also indicating that the C-terminal is disordered.

The C-terminus appears flexible, also in the ^15^N relaxation analysis, while the well-folded part of the sequence (residues 4–81) appears rigid. For the flexible C-terminus (relaxation data from residues 87–98) the extended model free model (Clore et al. 1990), i.e. model 4, is preferred. The internal correlation times are several hundred picoseconds and generalized squared order parameters for the slow internal component, i.e. S_s_^2^ are below 0.33 and the fast components, S_f_^2^, are in the range of 0.71–0.81. For the well-folded part the F-test indicates that model 1 is preferred with S^2^ in the range 0.74–0.96. The turn connecting helices 2 and 3 displays lower S^2^, while other turns appear as rigid as the elements of secondary structure on the picoseconds to nanosecond timescale. For residues T4 and F5 in the N-terminus, the model-free model (Lipari and Szabo 1982), i.e. model 2, is preferred with order parameters of 0.83 or higher and internal correlation times in the tens of picoseconds regime. For four residues (N51, I52, F65 and M66) the F-test indicates that significant exchange broadening contributes to R2 (the exchange contributions to R2 are 11, 13, 20 and 16 s^−1^ for N51, I52, F65 and M66, respectively). It is noteworthy that the local structures of the residues displaying exchange broadening are somewhat different in the crystal and solution structures. XTLSSR (King and Johnson, 1999) identifies N51 and I52 as members of a 3_10_-helix in 23 % of the members of the ensemble, while they are classified as α-helical in the other ensemble members. In the crystal structure (2xcj) XTLSSR classifies them as α-helical. Residues F65 and M66 are classified as hydrogen bonded turn in most ensemble members, while they are classified as α-helical in 18 % of the members. In the crystal structure they are classified as α-helical. It is tempting to hypothesis that the observed uncertainties in the local structure for those sites are genuine features of the protein as they are consistent with exchange broadening.

The observable HSQC-peaks in the complex with DNA demonstrate that the C-terminus remains flexible also in the presence of DNA. This may be somewhat surprising as the C-terminus displays significant sequence identity to C-proteins from related phages (Massad et al. 2010). However, the finding that the C-terminus is flexible also in the complex with DNA explain an in vitro activity assays which demonstrated that a C-terminally truncated variant P2 C (1–90) is capable of binding to the target DNA and function as a repressor of a reporter gene (Massad et al. 2010).

A few signals were tentatively assigned to residues part of the helix bundle (T55, L81 and A82). If correctly assigned, this would indicate a significantly increased flexibility of those residues upon binding to DNA. The absence of HSQC peaks from the rigid core of the protein is caused by rapid relaxation, likely caused by slow tumbling of the 66 kDa complex and hence provides some evidence in support of the tetrameric binding model. However, exchange between complexes with different stochiometry could lead to signal loss through exchange broadening, and cannot be completely ruled out based on the current data. Solving the crystal and solution structures of P2 C has opened way for many questions regarding how a small protein like P2 C can bind such long DNA stretch (the center-to-center distance between O1 and O2 half sites is 22 bp). In addition, having the C-terminus very flexible even upon binding the DNA raises questions regarding its biological role.
